# Association and correlation between stress, musculoskeletal pain and resilience in nurses before hospital accreditation maintenance assessment*

**DOI:** 10.1590/1518-8345.4658.3465

**Published:** 2021-08-30

**Authors:** Deise Juliana Rhoden, Christiane de Fatima Colet, Eniva Miladi Fernandes Stumm

**Affiliations:** 1 Universidade do Noroeste do Estado do Rio Grande do Sul, Ijuí, RS, Brazil.; 2 Scholarship holder at the Coordenação de Aperfeiçoamento de Pessoal de Nível Superior (CAPES), Brazil.

**Keywords:** Nurses, Psychological Resilience, Occupational Stress, Cortisol, Musculoskeletal Pain, Hospital Accreditation, Enfermeiras e Enfermeiros, Resiliência Psicológica, Estresse Ocupacional, Cortisol, Dor Musculoesquelética, Acreditação Hospitalar, Enfermeras y Enfermeros, Resiliencia Psicológica, Estrés Laboral, Cortisol, Dolor Musculoesquelético, Acreditación de Hospitales

## Abstract

**Objective:**

to associate and correlate musculoskeletal pain, stress and resilience of nurses in the maintenance of Hospital Accreditation Certification.

**Method:**

longitudinal study in two moments, before and after the Accreditation maintenance visit, March and June 2019, with 53 nurses from a hospital institution. The data collected was: sociodemographic, clinical and occupational variables, stress, osteomuscular pain and resilience. Descriptive variables, Chi-square test, t test, Fisher’s exact test, Pearson’s correlation and Spearman’s correlation coefficient were used.

**Results:**

most of the study participants had average stress levels before and after the evaluation. Most of those who reported pain were at medium stress levels at both times. The resilience capacity increased after the evaluation, which demonstrates that the experienced stressors were adequately addressed. There was no significant association between the cortisol levels and the perceived stress.

**Conclusion:**

occupational stress and musculoskeletal pain were experienced by nurses during the Accreditation processes. It was evident that individuality permeated the perception of stress and resilience allowed to overcome the tensions experienced. The study identified that there is a need for planning and implementation of actions to collaborate with the nurses in the best confrontation, aiming to promote resilience.

## Abstract (pt)

**Objetivo:**

associar e correlacionar dor musculoesquelética, estresse e resiliência dos enfermeiros na manutenção da Certificação de Acreditação Hospitalar.

**Método:**

estudo longitudinal em dois momentos, antes e depois da visita de manutenção da Acreditação, março e junho de 2019, com 53 enfermeiros de instituição hospitalar. Os dados coletados foram: variáveis sociodemográficas, clínicas e ocupacionais, estresse, dor osteomusculares e resiliência. Foram utilizadas variáveis descritivas, teste Qui-Quadrado, teste t, teste exato de Fisher, correlação de Pearson e coeficiente de correlação de Spearman.

**Resultados:**

a maioria dos participantes do estudo apresentou médios níveis de estresse, antes e depois da avaliação. A maioria dos que referiram dor encontrava-se em médio nível de estresse, nos dois momentos. A capacidade de resiliência aumentou depois da avaliação, o que demonstra que os estressores vivenciados foram enfrentados de maneira adequada. Não houve associação significativa entre os níveis de cortisol e o estresse percebido.

**Conclusão:**

o estresse ocupacional e a dor musculoesquelética foram vivenciados pelos enfermeiros durante os processos de Acreditação. Evidenciouse que a individualidade permeou a percepção do estresse e a resiliência permitiu superar as tensões vivenciadas. O estudo permitiu identificar que há necessidade de planejamento e implementação de ações para colaborar com os enfermeiros no melhor enfrentamento, visando promover a resiliência.

## Abstract (es)

**Objetivo:**

asociar y correlacionar el dolor musculoesquelético, estrés y resiliencia, de los enfermeros, en el mantenimiento de la Certificación de Acreditación Hospitalaria.

**Método:**

estudio longitudinal en dos momentos, antes y después de la visita de evaluación del mantenimiento de Acreditación Hospitalaria, en marzo y junio de 2019, en 53 enfermeros, de una institución hospitalaria. Los datos recogidos fueron: variables sociodemográficas, clínicas y laborales, estrés, dolor musculoesquelético y resiliencia. Fueron utilizadas variables descriptivas, test Chi-cuadrado, test t, test exacto de Fisher, correlación de Pearson y coeficiente de correlación de Spearman.

**Resultados:**

la mayoría de los participantes del estudio presentó niveles medios de estrés, antes y después de la evaluación. La mayoría de los que indicaron dolor se encontraba en el nivel de estrés medio, en los dos momentos. La capacidad de resiliencia aumentó después de la evaluación, lo que demuestra que los factores de estrés experimentados fueron enfrentados de manera adecuada. No hubo asociación significativa entre los niveles de cortisol y el estrés percibido.

**Conclusión:**

el estrés ocupacional y dolor musculoesquelético fueron experimentados por los enfermeros, durante los procesos de Acreditación. Se evidenció que la individualidad impregnó la percepción del estrés y la resiliencia permitió superar las tensiones experimentadas. El estudio identificó la necesidad de planificar e implementar acciones para colaborar con los enfermeros a mejorar el enfrentamiento, con el objetivo de promover la resiliencia.

## Introduction

The Hospital Accreditation is a modality of evaluation based on the quality and safety of the assistance in health institutions, according to established standards, with focus on permanent education and opportunities for improvement^(1)^. The interest of health institutions in seeking national or international certifications grows as health challenges become more complex and dynamic. Health care organizations tend to be successful when they adopt the posture of a culture of collaboration, flexibility and risk management, focusing on quality, safety and sustainability^(2)^.

Nurses play an important role in the success of institutions seeking Accreditation Certifications, because the characteristics of the profession allow them to empower themselves in the work processes and in the construction of evidence that supports this methodology. This possibility, however, requires an attentive look at the health of these professionals due to the demands inherent to this process, more specifically, tension and overload^(3)^. In this sense, occupational stress is a process in which the professional interprets the demands/work requirements as stressors, which exceed their ability to overcome them; therefore compromising their health^(4)^.

Stress unbalances the organism and is controlled by the Autonomous Nervous System (ANS), by external and internal stimuli. The phases of stress evolution are: alarm, resistance and adaptation and exhaustion, which occurs in more serious situations, with repercussions in several systems of the human body^(5)^. In prolonged exposure to stress, the hypothalamus-hypophysis-adrenal axis (HPA) and ANS stimulate hormones, among them cortisol, which, when in excess, damages the immune system^(6)^. In this sense, cortisol is an important biomarker of stress^(6-7)^.

Stress triggers in the body reactions similar to toxicchemical substances, related to the loss of homeostasis in various systems of the human body^(8)^. Thus, stress is associated with musculoskeletal pain perceived by nurses in the face of stressors^(8-9)^. The adversities faced by these professionals, in their daily work, are related to physical and psychic loads, which constitute health risks and alter their perception of pain^(9)^. Musculoskeletal pain and stress are individual sensations that emerge as a result of the subjectivity of the individual^(10)^.

These considerations refer to the need for the professional to develop psychic and physical characteristics that prepare him/her to be resilient in the face of adversities in the work environment^(11)^. Resilience, in the field of psychology, is the person’s capacity to deal with problems, adapt to changes, resist pressures and overcome adversities, without suffering any psychological, emotional or physical damage with the use of coping strategies^(12)^. In this context, the expansion of resilience capacity may contribute to the facing of adversities by nurses, in order to overcome the stressors that emerge from Hospital Accreditation Certification audits and, also, the musculoskeletal pains mentioned by them, which may also be associated with the pressures experienced during that period.

The construction of this study was based on considering the stress triggered by the Hospital Accreditation process, its potential to cause pain and its capacity to be modulated by resilience, allied to the knowledge gap in this area. Auditing is a process experienced by nurses, concomitant with the demands and requirements already existing in their daily work. In this sense, this activity, as another stressful moment for these professionals, overloads them in their activities, exposing them to illness. With the study, we sought to know how nurses react to the stressors inherent to the process and, thus, contribute to the knowledge bases on hospital accreditation with the knowledge of these professionals on the subject and, also, we sought to allow nurses and managers to build strategies to maintain health and achieve institutional goals. Thus, this research aimed to associate and correlate musculoskeletal pain, stress and resilience of nurses in the maintenance of Hospital Accreditation Certification.

## Method

It is a longitudinal study of two moments and developed in a philanthropic hospital of size IV (hospital classification of the Unified Health System-UHS, Ordinance 2.224 of 2002), classified as high complexity in traumatology, neurology and obstetrics and located in the Northwestern Region of the State of Rio Grande do Sul (RS), Brazil. The choice for the institution was intentional due to the fact that it was going through an Accreditation audit process, being this the object of the present work, in order to continuously seek the improvement of its services and demonstrate concern with its professionals.

All 68 nurses from the above mentioned institution were invited to participate in the study, including those who work in different sectors of this institution, of both sexes, regardless of the time spent working in the place researched; those who were on vacation, certificate or maternity leave, pregnant women, in treatment with corticosteroids and with a diagnosis of Addison’s disease were excluded due to the fact that they present alterations in the physiological levels of cortisol. After the inclusion and exclusion criteria were applied, 53 nurses were left to participate in the study.

The variables analyzed in the study were: sociodemographic, occupational, clinical, stress, musculoskeletal pain, resilience and salivary cortisol.

The following instruments were used for data collection:

1. Questionnaire elaborated by the researchers, composed of eight (8) questions with sociodemographic, occupational and clinical variables, which correspond to: date of birth, marital status, children, sex, work shift, work load, if you have another job, if you have any disease and if you use continuous medication. The same was evaluated through a pilot study in which six (6) nurses participated. After the test, the changes were unnecessary.
2. The Bianchi Stress Scale (BSS)^(13)^ was built and validated for Portuguese in 2009. It integrates categorization data, with 51 items that describe activities of the nurse’s work, gathered in six domains: Domain A - Relationship with other units and supervisors; Domain B - Proper operation of the unit; Domain C - Personnel administration; Domain D - Nursing assistance provided to the patient; Domain E - Coordination of activities; Domain F - Working conditions. The stress level of the nurse results from the sum of these 51 items, scored on a Likert scale, from 0 to 7. The value zero is attributed when the nurse does not execute the activity, 1 considers the activity little exhausting, 4 medium and 7 highly exhausting^(13)^. Also, on this scale, to verify the total score, the sum varies from 51 (when the nurse points out as not very stressful all the activities) to 357 points (7 points, when very stressful) and as for the total of responses, zero corresponds to no stress level; from one to 119 corresponds to a low stress level; from 120 to 238, medium and from 239 to 357, high stress level^(14)^.
3. The Nordic Osteomuscular Symptoms Questionnaire (NOSQ) was translated and validated into Portuguese in 2002 was used to identify the occurrence of musculoskeletal symptoms in nurses. It is a selfapplicable instrument, with answers “YES” or “NO”, according to the participants’ perception of the symptoms and their work activity, in ten anatomical regions, 12 months and seven days prior to the evaluation^(15)^. The authors stress that the respective instrument is restricted to the occurrence of musculoskeletal symptoms without emphasizing severity or frequency.
4. Nurses’ resilience was evaluated through the Resilience Scale (RS), developed based on the Resilience Scale in 1993, translated and validated into Portuguese^(16)^. It verifies the level of positive psychosocial adaptation of the individual to important life situations. The instrument includes 25 items, on a Likert scale: 1 (I totally disagree), 2 (I disagree a lot), 3 (I disagree little), 4 (I neither agree nor disagree), 5 (I agree little), 6 (I agree a lot) and 7 (I totally agree). The sum of each item varies between 25 points for low resilience and 175 points for high resilience^(16)^ and are classified into: low resilience, score below 121; medium resilience, score from 121 to 146 and high resilience, score above 147^(17)^. The RS comprises three factors: Factor I - 14 items related to stock and value resolutions (described in items: 2, 6, 8, 10, 12, 14, 16, 18, 19, 21, 23, 24 and 25); Factor II - 6 items, characterized by independence and determination (described in items: 5, 7, 9, 11, 13 and 22) and Factor III - 5 items, characterized by self-confidence and ability to adapt to situations (described in items: 3, 4, 15, 17 and 20).

To evaluate the physiological stress of the nurses, salivary cortisol was collected based on three saliva samples. Nurses working in the morning and afternoon shifts collected the samples when they woke up, one hour after starting their work and one hour before ending their shift. Nurses working at night collected the samples when they woke up, on the day they would work and had not worked the night before and the other samples, one hour after starting their work and one hour before ending their shift. They were given a kit containing three Salivette tubes (Salivette^®^, Sarstedt, Germany) duly identified with the participant number and time of collection (1^st^, 2^nd^ or 3^rd^ collection), as well as guidance as to the stepby-step collection, necessary to avoid that losses or changes could render the sample unusable and, finally, the sample identification document for the laboratory. The written information provided referred to: eating a light diet at breakfast; not eating food, beverages (except water) or smoking for thirty minutes before the collection; washing the mouth with light cheeks before performing the collection; not performing the collection if there are oral lesions with the presence of active or potential bleeding; not having undergone dental treatment in the last 24 hours before the collection; not brushing the teeth in the last three hours before the collection in order to avoid gum bleeding and not having ingested alcohol in the previous 12 hours^(18)^. The choice of cortisol evaluation via saliva was considered so that the patient could collect his/her own samples at the most appropriate time for the study, without the need for intervention of a professional, puncture or displacement to a laboratory.

To obtain the sample, each nurse was directed to open the Salivette, remove the swab and position it under the side of the tongue to stimulate salivation for 3 to 5 minutes. After realizing that it was saturated with saliva, he was instructed to remove it from the mouth and insert it again in the Salivette tube, closing it firmly. After collecting the saliva, the material was stored refrigerated at 2°C to 8°C in thermal containers indicated for collectors and sent to Northwestern Regional University of Rio Grande do Sul -UNIJUÍ’s clinical analysis laboratory, with careful observation as to storage, transport and analysis of the samples. The reference values for salivary cortisol were made available by the support laboratory, which analyzed the samples of this study being: from 6 h to 10 h - <0.78 ug/dL, from 16 h to 20 h - <0.24 ug/dL and from 23 h 30 min to 00 h 30 min - <0.20 ug/dL; the cortisol was determined by the electrochemical analysis method, considered the gold standard method for this result.

The data collection took place in two different moments, before and after the evaluation for Level 2 Hospital Accreditation Certification, with the use of all the above mentioned instruments. The first collection took place in March 2019, the week before the audit visit. The second collection took place in the first two weeks of June 2019, 60 days after the audit visit. This period, between the first and the second collection, was stipulated by the researchers in order to wait for the receipt of the audit report with the result of it and the due notes made by the auditors. This period also allowed these results to be passed on to the nurses and they had received this feedback from the management, as well as guidelines on strategies for implementing suggestions and pending. Among the notes raised by the auditors, it should be noted that the result of the audit was positive, the main demands and adjustments pointed out refer to issues of infrastructure and interaction of processes.

The data collection in both moments was carried out by the researchers with the help of three a, academics from the Nursing course, duly trained regarding the study objectives and application of the instruments. The collection occurred in an individual way, filled by the researcher during the work period of the participants, in the premises of the institution. After delivery, the instruments were checked for inconsistencies. The nurses were then instructed how to proceed with the collection of saliva samples.

The data that integrates this research constituted a database with double typing that, with the help of the software Statistical Package for the Social Sciences (SPSS), version 17.0 through which they were analyzed. The descriptive variables were displayed with absolute (n) and relative (%) frequencies (lower and upper limit, mean, standard deviation and coefficient of variation). The Kolmogorov-Smirnov normality test was applied. To cross the variables, it was considered the significant association p<0.05, the following tests were used: Chi-square test to associate: resilience and moment of collection of cortisol; t test to associate resilience and moment of collection of cortisol (not significant p>0.05); Fisher’s exact test associated total stress level of the Bianchi Stress Scale and musculoskeletal symptoms; Pearson’s correlation to verify the degree of correlation between the domains of the Bianchi Stress Scale, resilience and cortisol.

The study was approved by the Research Ethics Committee with opinion no. 3,085,366, CAAE no. 03781418.1,0000.5322.

## Results

Fifty-three nurses participated in this survey, the majority (75.5%) female and 58.5% with an average age between 31 and 40 years old, 83% said they lived with a partner and 66% of them had children. The participants had worked in the health care institution in an average of 9.5 years, 52.8% in the morning and afternoon shifts, 20.8% at night and 26.4% without a fixed shift. More than half (62.3%) supervised the activities of the units, represented by care activities to the patient, 20% of them aggregated the coordination function with care, the others (17%) act exclusively as coordinators. In relation to the hourly load of these professionals, more than half (54.7%) worked 36 weekly hours, 39,6% more than 36 hours and the others worked a lesser day.

The data in Table 1 explains the diseases of the nurses participating in the study. Most did not use continuous medication and stated that they did not have any disease. Of those who reported having a disease, the highest percentages were related to hypertension, heart disease, spondyloarthropathy and depression.

**Table 1:** Health characteristics of nurses (n=53) working in a philanthropic hospital in the Northwestern Region of the State of RS, Brazil, 2019

Characteristics		n*	%
Carrier of disease	Yes	7	13.2
	No	46	86.8
Total		53	100.0
Which	Depression	1	14.2
	Spondyloarthropathy	1	14.2
	SAH^†^	4	57.1
	SAH^†^, Heart disease	1	14.2
Total		7	100.0
Use of medication	Yes	20	37.7
	No	33	62.3
Total		53	100,0

* n = Number; ^†^SAH = Systemic arterial hypertension

Sequentially, in Table 2, the stress perceived with the use of BSS and the resilience of the nurses participating in the study, before and after the evaluation of maintenance of the Hospital Accreditation were related. Based on this association, it was possible to affirm that a greater number of nurses were at average levels of stress and resilience, before and after the assessment.

The high percentages of those with high resilience, 26.4% before the evaluation and 35.8% after the evaluation, with emphasis on the fact that there was an increase in resilience after the evaluation.

Also, regarding the data contained in Table 2, the result showed that there is no statistically significant association between perceived stress and resilience (p>0.05) of professionals at the two moments they were evaluated.

**Table 2:** Frequency of resilience according to the total stress levels of the Bianchi Stress Scale (BSS) in nurses (n=53) working in a philanthropic hospital, before and after the evaluation of maintenance of the Hospital Accreditation Certification - March and June. Northwestern Region of the State of RS, Brazil, 2019

Moment of collection	Bianchi Stress Scale (BSS)	Resilience				
		Low	Medium	High	Total	p-value
BA* ^†^	Low	5(9.4)	10(18.9)	7(13.2)	22(41.5)	0.228
	Medium	3(5.7)	21(39.6)	7(13.2)	31(58.5)	
	Total	8(15.1)	31(58.5)	14(26.4)	53(100)	
AA* ^‡^	Low	3(5.7)	7(13.2)	10(18.9)	20(37.7)	0.247
	Medium	7(13.2)	17(32.1)	9(17.0)	33(62.3)	
	Total	10(18.9)	24(45.3)	19(35.8)	53(100)	

*Non-Significant Chi-square Test p>0.05; †BA = Before Hospital Accreditation Certification maintenance assessment; ‡AA = After Hospital Accreditation Certification maintenance assessment

The resilience scale comprises three factors and, in Table 3, the descriptive statistics of the scores were applied to each one, divided according to the stress levels in which the participants were and the difference between the mean of those with low and medium stress levels was verified. With this test, it was verified that there was no statistically significant difference between participants’ stress levels in each of the factors of the Resilience Scale, at the two moments of assessment.

**Table 3:** Resilience Statistics according to the total stress levels of the Bianchi Stress Scale (BSS*) in nurses (n=53) working in a philanthropic hospital, before and after the evaluation of maintenance of the Hospital Accreditation Certification - March and June. Northwestern Region of the State of RS, Brazil, 2019

Moment of collection	Resilience	BSS^*^ Level of Stress	Descriptive statistics						Test t p-value
			n^†^	LL^‡^	UL^§^	Mean	SD^||^	CV^¶^(%)	
BA^**^	Factor 1	Low	22	63	96	82.50	6.96	8.44	0.241
		Medium	31	69	90	80.52	5.23	6.50	
		Total	53	63	96	81.34	6.03	7.41	
	Factor2	Low	22	11	37	25.45	6.74	26.47	0.317
		Medium	31	18	34	26.97	4.17	15.45	
		Total	53	11	37	26.34	5.38	20.42	
	Factor3	Low	22	9	34	28.50	5.29	18.56	0.149
		Medium	31	25	35	30.16	2.91	9.65	
		Total	53	9	35	29.47	4.11	13.94	
	Total	Low	22	114	166	136.45	14.04	10.29	0.716
		Medium	31	115	154	137.65	9.64	7.00	
		Total	53	114	166	137.15	11.56	8.43	
AA^††^	Factor 1	Low	20	70	95	83.30	7.49	8.99	0.283
		Medium	33	65	94	81.09	7.00	8.63	
		Total	53	65	95	81.92	7.20	8.79	
	Factor2	Low	20	22	40	27.35	4.74	17.32	0.933
		Medium	33	9	42	27.21	6.35	23.33	
		Total	53	9	42	27.26	5.75	21.07	
	Factor3	Low	20	24	33	29.55	2.63	8.88	0.429
		Medium	33	19	34	28.88	3.16	10.94	
		Total	53	19	34	29.13	2.96	10.17	
	Total	Low	20	117	165	140.20	12.68	9.04	0.422
		Medium	33	109	165	137.18	13.43	9.79	
		Total	53	109	165	138.32	13.11	9.48	

*BSS = Bianchi Stress Scale; †n = Number; ‡LL = Lower Limit; §UL = Upper Limit; ||SD = Standard Deviation; ¶CV = Coefficient of Variation; **BA = Before the evaluation of maintenance of the Hospital Accreditation Certification; ††AA = After the evaluation of maintenance of the Hospital Accreditation Certification. Resilience: Factor I (Resolutions of actions and values); Factor II (Independence and determination) and Factor III (Self-confidence and ability to adapt to situations). Non-significant p>0.05

Before the assessment for the maintenance of the varied little in relation to the low and medium level of stress (82.5 for low level and 80.52 for medium level of stress). It was also evident that the Coefficient of Variation was 8.44 and 6.50, respectively, which demonstrated a small oscillation in the value of the resilience score for each participant. In the other factors, the values referring to the coefficient of variation were also below 30%, which showed that there was homogeneous variability of the resilience scores in relation to the average of the same, for all nurses.

**Table 4:** Total Stress Level of the Bianchi Stress Scale (BSS*) according to musculoskeletal symptoms in the last 7 days referred by nurses (n=53) working in a philanthropic hospital, before and after the evaluation of maintenance of the Hospital Accreditation Certification - March and June. Northwestern Region of the State of RS, Brazil, 2019

Osteomuscular symptoms		Before Accreditation			After Accreditation		
		BSS^*^- Level of Stress			BSS^*^- Level of Stress		
		Low	Low	Low	Low	Médio	**p-valor^†^**
Neck	No	16(30.2)	19(35.8)	0.557	15(28.3)	19(35.8)	0.247
	Yes	6(11.3)	12(22.6)		5(9.4)	14(26.4)	
Shoulders	No	15(28.3)	14(26.4)	0.161	14(26.4)	17(32.1)	0.253
	Yes	7(13.2)	17(32.1)		6(11.3)	16(30.2)	
Upper back	No	15(28.3)	14(26.4)	0.161	15(28.3)	19(35.8)	0.247
	Yes	7(13.2)	17(32.1)		5(9.4)	14(26.4)	
Elbows	No	22(41.5)	29(54.7)	0.505	20(37.7)	29(54.7)	0.285
	Yes	-	2(3.8)		-	4(7.5)	
Wrists or hand	No	20(37.7)	24(45.3)	0.277	20(37.7)	30(56.6)	0.282
	Yes	2(3.8)	7(13.2)		-	3(5.7)	
Lower back	No	18(34.0)	22(41.5)	0.520	16(30.2)	23(43.4)	0.527
	Yes	4(7.5)	9(17.0)		4(7.5)	10(18.9)	
Hip/thighs	No	21(39.6)	23(43.4)	0.064	17(32.1)	24(45.3	0.500
	Yes	1(1.9)	8(15.1)		3(5.7)	9(17.0)	
Knees	No	18(34.0)	28(52.8)	0.431	18(34.0)	28(52.8)	0.697
	Yes	4(7.5)	3(5.7)		2(3.8)	5(9.4)	
Ankles/feet	No	20(37.7)	23(43.4)	0.116	18(34.0)	29(54.7)	0.999
	Yes	2(3.8)	8(15.1)		2(3.8)	4(7.5)	

* BSS = Bianchi Stress Scale;^†^Fisher’s exact test, significant ratio for p<0.05

Table 4 shows the relationship between the musculoskeletal symptoms reported in the last seven days, with stress levels perceived by nurses responding to BSS in both moments, before and after the assessment for maintaining Hospital Accreditation Certification. In this one it was verified that, between the pain referred to in the different anatomical regions, when related to the stress levels (low and medium), there was no statistically significant association between them, in the two moments of assessment.

Also, in Table 4, regarding the frequencies of musculoskeletal pain and stress according to the BSS, in all anatomical regions that the instrument contemplates, most of those who reported pain were at medium level of stress, at both times of evaluation.

Finalizing the data presentation, Table 5 presents the correlation of the BSS domains, resilience and salivary cortisol levels in the three collection times in the two periods of this survey, before and after the evaluation for the maintenance of the Hospital Accreditation Certification. The results allow us to state that there was no correlation between these variables.

**Table 5:** Correlation coefficient of the domains of the Bianchi Stress Scale (BSS*), resilience and cortisol in nurses (n=53), before and after the maintenance visit of the Hospital Accreditation Certificate, working in a philanthropic hospital - March and June. Northwestern Region of the State of RS, Brazil, 2019

Domains BSS^*§§§^		**Cortisol BA^†^**				**Cortisol AA^‡^**			
		**WW^§^**	**BW||**	**EW^¶^**	R**	**WW^§^**	**BW||**	**EW^¶^**	R**
A^††^	R	0.183	-0.034	0.150	-0.052	0.016	0.054	0.022	-0.096
	P	0.190	0.806	0.284	0.712	0.911	0.702	0.876	0.494
B^‡‡^	R	0.124	-0.228	-0.003	0.031	0.068	0.158	-0.208	0.037
	P	0.375	0.100	0.982	0.824	0.630	0.259	0.134	0.791
C^§§^	R	-0.008	-0.099	-0.084	0.144	0.149	-0.219	0.001	-0.011
	P	0.952	0.482	0.550	0.303	0.287	0.116	0.996	0.935
D^***^	R	0.016	-0.079	0.051	-0.035	-0.105	-0.100	0.027	-0.072
	P	0.907	0.574	0.718	0.804	0.463	0.485	0.851	0.613
E^†††^	R	-0.083	-0.048	0.091	-0.037	0.050	0.078	-0.109	-0.147
	P	0.556	0.730	0.519	0.791	0.725	0.578	0.437	0.294
F^‡‡‡^	R	0.069	-0.138	0.114	-0.144	0.040	-0.094	-0.139	-0.263
	P	0.621	0.323	0.416	0.304	0.778	0.502	0.320	0.057
Total	R	0.085	0.001	-0.062	-0.020	0.033	-0.051	-0.006	-0.099
	P	0.545	0.992	0.659	0.886	0.812	0.716	0.963	0.482
R^**||||^	R	-0.056	0.055	0.078	1	0.091	-0.031	-0.072	1
	p-valor	0.689	0.697	0.580	-	0.516	0.824	0.606	-

^*^BSS = Bianchi Stress Scale; ^†^BA = Before the evaluation of maintenance of the Hospital Accreditation Certification; ^‡^AA = After the evaluation of maintenance of the Hospital Accreditation Certification; ^§^WW = When waking up; ^||^BW = Beginning of work; ^¶^EW = End of work; ^**^R = Resilience; ^††^A = Relationship with other units and supervisors; ^‡‡^B = Activities related to the proper functioning of the unit; ^§§^C = Activities related to personnel administration; ^***^D = Nursing assistance provided to the patient; ^†††^E = Coordination of unit activities; ^‡‡‡^F = Working conditions for the performance of the activities of the nurse. Significant correlation with p<0.05; ^§§§^Spearman correlation; ^||||^Pearson correlation. Domain scores: value 1 when finding the activity “not very stressful”; 4 for “medium”; 7 for “very stressful” and 0 in case “does not perform the activity”

Also, in Table 5 Pearson’s correlation test showed that the means did not have the same behavior, which indicated that each individual responded differently to the stress experienced in the work environment.

## Discussion

The population studied comprised 53 nurses, predominantly female, a characteristic of nursing and also evidenced in other studies^(19-21)^. By relating this characteristic of the population studied to stress, it is observed that women are more exposed to stress due to the overload of domestic and professional chores^(19)^. The majority of the participating nurses of the study acted as supervisor of the activities of nursing, however, an expressive percentage of these professionals accumulated activities of unit coordination with care activities. It’s up to the unit’s nurse coordinator to manage the nursing assistance with activities directed to the personnel management, such as: elaborate scales, trainings, selection of personnel and, thus, take care that the nursing team has favorable conditions to give assistance to the patients^(21)^. It is worth noting that the various requirements necessary to maintain Hospital Accreditation Certification are added to the responsibilities of the coordinating nurse, with the management of welfare indicators, costs and revenues of the unit and the formulation and restructuring of work protocols^(22)^.

Most of the nurses who participated in this research presented good health conditions, however, some diseases were mentioned, such as hypertension, heart diseases, depression, spondyloarthropathies and these may be related to chronic exposure to occupational stress. It is important to emphasize that high demands stimulate the occurrence of some diseases due to the increase in demand associated with little control of the professional over his work activity. When the professional maintains high levels of stress, for a long period of time, the tension on the central nervous system and cardiovascular system increases^(19)^.

The processes that integrate Hospital Accreditation have positive aspects such as people development, qualification, safety and standardized work processes. However, there are negative impacts such as stress, pressure with goals and deadlines, higher charges on nurses and lack of appreciation, which were also feelings experienced by professionals during this process^(23)^ and such factors can cause stress. This, when in low doses, can be beneficial to the health of the nurse, resulting in disposition, interest, enthusiasm. However, when in higher doses, it is harmful and manifests itself from some symptoms, such as fatigue, irritability, inattention, which harms him in his work. The occupational stress in the daily routine of the nurse can come from several aspects of his work activity and with implications in personal life^(4)^. In this sense, high workloads, pressure with time and deadlines and the stress of dealing with lives contribute to professional exhaustion^(24)^.

Most participants in this study perceived their work activities as stressful, but at a medium level, this result deserves attention and actions aimed at the risk of compromising their health and the quality of the assistance provided. The altered stress levels, for long periods, excite the organism, which increases the tension of the cardiovascular and nervous systems and exposes the professional to sickness^(19)^. A study conducted with Iranian nurses, in order to verify their perception of the impacts of Accreditation, concluded that even in the face of numerous demands, it has a positive impact on the quality of education assistance and professional growth^(25)^. In a study conducted with Jordanian nurses, it was concluded that Hospital Accreditation is a stressful process for these professionals and that the stress levels were higher before the assessment visit^(26)^.

The analysis of the high resilience of nurses participating in the research is a positive and important factor for the maintenance of health, prevention of damage and quality of care, since resilience helps professionals to resist the experience of stressful situations and also to support and face changes. A study evaluated the impact of a resilience program implanted in a hospital in the United States, specific for the nursing team that worked in the Pediatric Intensive Care Unit (PICU) and the authors concluded that the program presented positive results and the actions implemented, focused on increasing the resilience of the professionals, were effectives^(27)^. In the complexity of human beings and their work, in facing the adversities present in the work context, personal and environmental resources must be continuously promoted, because resilience is a “being” and not a “resilient being”^(28)^.

It is understood that the fact that there was no significant relationship between stress perceived by professionals, according to the total stress levels of BSS and resilience, does not mean that they are less important results, as well as the resilience of nurses has increased after the evaluation process for maintaining Hospital Accreditation Certification. These results demonstrate that the stressors experienced by nurses before the evaluation were adequately addressed, i.e., concomitantly there was an increase in resilience capacity after the evaluation, another positive factor for the proper facing of the process to which they were subjected. A study conducted with nurses, with the objective of investigating the role and influence of nursing in the hospital Accreditation process in a private hospital in Belo Horizonte, Minas Gerais, Brazil, identified that the feeling of pride and satisfaction is present in nurses because they feel responsible for achieving institutional results and relate such results to their professional growth^(23)^. Another study points out the high resilience as a positive factor for nurses, since they associate, in their work, greater resilience with their low turnover^(29)^.

Regarding the pain symptoms reported by survey participants, the fact that most who reported pain are at medium stress levels is an indicator of the damage that stress can cause to workers’ health. The presence of emotional wear and tear related to the work that the professional performs is associated with the occurrence of stress^(30)^. The stress, experienced at work, goes through psychological aspects and encompasses physical issues of the individual such as musculoskeletal pain. The tensions experienced by nurses in their work environment are referred to as important factors of impact on pain^(8)^. In the meantime, it is possible to characterize pain as a sign of stress, when susceptible individuals are constantly faced with stressors and tense situations and in response, develop musculoskeletal pain^(31)^. A study conducted with nurses from Southeast Asia concluded that these professionals faced high demands of work, stress, fatigue and musculoskeletal pain in the neck, shoulder, upper and lower back and foot region^(32)^.

When nurses are exposed to different stressors and tensions in their work environment they may present psychological symptoms such as: negative emotions, anxiety and tensions. Increased muscle tension affects hormonal, circulatory and respiratory reactions, which contributes to the occurrence of musculoskeletal pain^(24)^. In this sense, the musculoskeletal pain identified by the participants of this study resulted from a negative outcome of the stress experienced, indicated by the validated instrument applied in this study. Stressful situations experienced at work are related to musculoskeletal pain. A study conducted with Icelandic unit nurses concluded that daily work stress had any strong correlation with musculoskeletal pain and the places where these professionals most reported pain were in the neck and shoulder areas^(33)^.

Regarding the physiological stress, verified in this study by salivary cortisol, although without significant associations to the Accreditation and to the work moment, there was variability in the values found, which can be an indication of the individual response of these professionals before the stress factors experienced during the Accreditation process.

A study conducted with nurses from the United Arab Emirates also verified physiological stress, by salivary cortisol levels and correlated it with the stress perceived in the work routine and, likewise, no significant association was found between the variables, although the authors concluded that lower salivary cortisol levels were related to better coping mechanisms and also emphasized that individual coping strategies were important for the health of nurses^(34)^. There is also a greater number of studies that evaluate stress with the use of validated instruments^(15,19-20,25,28,33)^ and there are few studies of stress evaluation through cortisol with nursing professionals during the Accreditation process.

The condition of the nurses who participated in the research is worthy of actions and educational interventions in order to expand their knowledge and, thus, help them to face adversities in the work environment in an appropriate manner and, especially with the aim of increasing resilience capacity.

The limitations of the study include the fact that it was conducted in a single hospital, and some variables collected were self-referenced. It is worth mentioning that it made it possible to know the results of the nurses’ experiences regarding musculoskeletal pain, physiological and psychological stress and resilience and association between such variables, during the evaluation audit of maintenance of the Hospital Accreditation Certification and, thus, understand how these symptoms and feelings can influence the health of these professionals.

With the data found one can seek to develop actions to prevent this harmful impact to health, maintaining the quality results offered by the qualification. This study brought a new knowledge about a subject that needs to be explored a lot, considering the lack of investigations that approach this subject. New researches are still needed, multicentric, with larger samples for the search of causal inferences limited to this one in function of the size of the studied population.

## Conclusion

The occupational stress verified in this study occurred in the nurses who participate in the processes that integrate the Hospital Accreditation. It was verified that after the process of Hospital Accreditation there was an increase in resilience, as well as the presence of pain in different anatomical regions, without association to stress levels. Salivary cortisol values did not vary significantly during the collection moments, however, Pearson’s correlation test was used, which demonstrates variability in the means, related to distinct responses to stress experienced in the work environment in each of the participants.

The results point out possible health problems associated to stress and, in this sense, it is considered important the planning and implementation of actions by managers, with the purpose of helping them to obtain a better confrontation, without damages to health and to the quality of the work developed by them. Allied to these actions, it is important to help this professional category to increase its resilience capacity, a *sine qua non* condition for health maintenance and prevention of stress damages, many times irreparable, especially at times when the health professional finds himself with a higher level of responsibility and collection, such as during the Hospital Accreditation period.
